# Avelumab Treatment in Very Elderly People With Advanced Merkel Cell Carcinoma: A Case Report and Review of the Literature

**DOI:** 10.7759/cureus.97683

**Published:** 2025-11-24

**Authors:** Eleonora Cerchiaro, Rosalba Barile, Alessandro D'Aveni, Alessia Liguori, Giuseppe Di Lucca

**Affiliations:** 1 Department of Medical Oncology, ASST (Azienda Socio Sanitaria Territoriale) Valle Olona, Ospedale di Saronno, Saronno, ITA

**Keywords:** avelumab, case report, elderly cancer patients, immunotherapy, merkel cell carcinoma

## Abstract

Merkel cell carcinoma (MCC) is a rare and aggressive neuroendocrine skin cancer, with a higher incidence in elderly and immunocompromised patients. Immunotherapy with anti-programmed death-ligand 1 (anti-PD-L1) agents, particularly avelumab, has revolutionized the treatment landscape of advanced MCC. However, evidence regarding its safety and efficacy in very elderly patients (aged ≥85 years) remains scarce. We report the case of a 95-year-old woman with locally advanced MCC treated with avelumab, achieving a major clinical response and maintaining disease control after treatment discontinuation. A literature review on immunotherapy in the very elderly MCC population is also provided.

## Introduction

Merkel cell carcinoma (MCC) is a highly aggressive skin cancer of neuroendocrine origin, associated with Merkel cell polyomavirus (MCPyV) and UV exposure. It predominantly affects older adults, with the median age at diagnosis being over 70 years. Immunotherapy with avelumab, an anti-PD-L1 monoclonal antibody, has become the standard first-line treatment for advanced MCC [1,2]. However, data on its use in the very elderly (aged ≥ 85 years) are extremely limited.

This report illustrates the clinical course and therapeutic response of a 95-year-old woman with advanced MCC treated with avelumab, highlighting the feasibility of immunotherapy in selected very elderly patients.

## Case presentation

**Figure 1:**
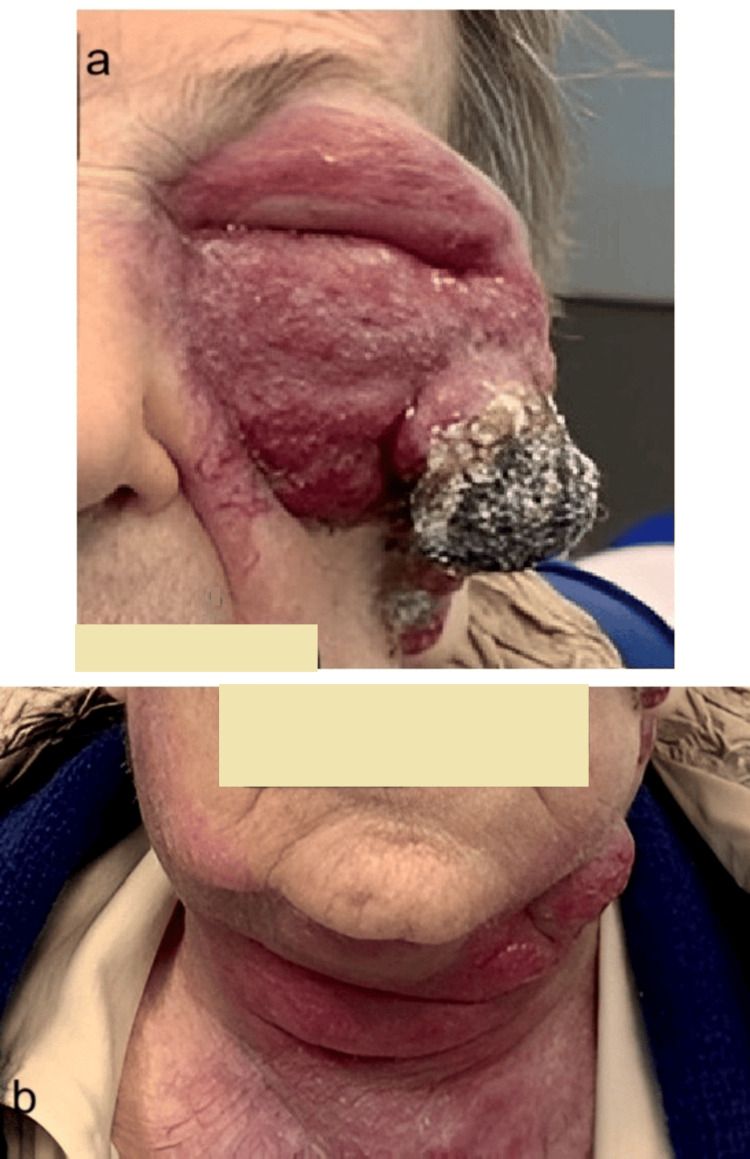
Patient at initial presentation (April 2024) (a) left hemi-face; (b) neck

A 95-year-old woman with no prior history of cancer presented in April 2024 with a rapidly growing suborbital mass involving her left hemi-face and extending to the neck (Figure 1). Histological examination confirmed Merkel cell carcinoma. MRI revealed a 6 cm multilobular hemorrhagic cutaneous lesion infiltrating the subcutaneous and periorbital soft tissues. She was partially dependent because of Parkinson's disease, but cognitively intact. No surgical or radiotherapeutic options were feasible due to the extent of disease and frailty.

Despite her advanced age and comorbidities, the patient was started on avelumab monotherapy (10 mg/kg every two weeks) after peripherally inserted central catheter (PICC) line placement and provision of adequate informed consent. Treatment was well tolerated, and no acute infusion reactions occurred with standard premedication (hydrocortisone 100 mg, acetaminophen 1000 mg, and oral cetirizine 10 mg during the first four infusions, as recommended).

**Figure 2:**
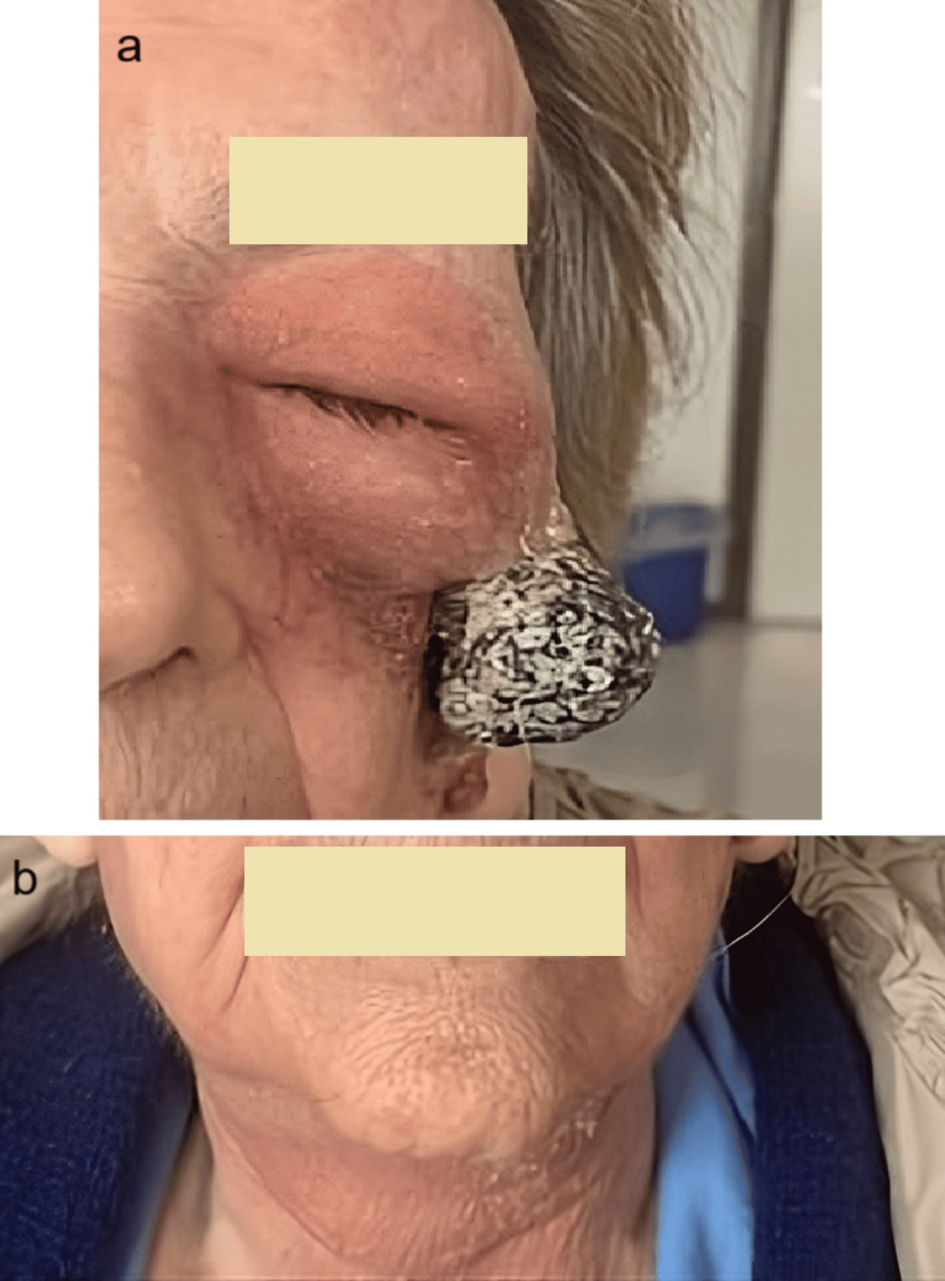
Disease response after seven months of treatment with avelumab (November 2024) (a) left hemi-face; (b) neck

From May to October 2024, she received 11 avelumab infusions and achieved evident, progressive tumor shrinkage. By November 2024, a major clinical response was documented, with only a small residual lesion (~5 mm) remaining (Figure 2). Her overall clinical condition remained stable throughout treatment. Discontinuation of therapy was agreed upon due to age and patient preference. At the last clinical assessment in February 2025, disease control was maintained, with no new lesions and a stable performance status. No immune-related adverse events were observed during treatment or at subsequent visits.

The most recent follow-up was in September 2025 through a telephone update from her son. The patient was alive, with no reported tumor regrowth, but had been transferred to a long-term care facility due to cognitive decline and worsening motor disability.

## Discussion

This case highlights the potential benefit of avelumab in a patient aged over 95 years, a population largely underrepresented in clinical trials. While immunosenescence and comorbidities pose challenges in this age group, our experience confirms that performance status and individual frailty are more informative than chronological age alone. 

Data from the JAVELIN Merkel 200 trial established avelumab as an effective treatment in metastatic MCC, with an overall response rate (ORR) of 31.8% in chemotherapy-refractory patients and durable responses in 82% of responders [1]. However, this study excluded patients with Eastern Cooperative Oncology Group Performance Status (ECOG PS) >1 and did not stratify efficacy or toxicity data specifically by age above 80 years. 

The expanded access program (EAP) analyzed 494 patients with metastatic MCC, including many who were not eligible for clinical trials [3]. The ORR was 46.7%, with a complete response in 22.9% of patients. Although age-specific outcomes were not reported, this broader, more inclusive dataset suggests that avelumab may also be beneficial in frail or older populations typically excluded from phase II trials. 

The four-year follow-up of the JAVELIN Merkel 200 part B study, which included treatment-naïve patients, confirmed long-term benefit with avelumab as first-line therapy, showing a median overall survival (OS) of 20.3 months and a four-year OS rate of 38% [2]. While patients over 90 were again not analyzed separately, the median age was >70 years, supporting efficacy in the elderly. 

A pooled safety analysis of avelumab across solid tumors from data of the Phase 1 JAVELIN trial reported a manageable profile, with grade ≥3 treatment-related adverse events (TRAEs) occurring in only 10.2% of patients [4]. Infusion-related reactions (IRRs) were typically low-grade and manageable with premedication, similar to the case described. Importantly, serious immune-related adverse events were rare (<2%). 

The association of older age with immune-related adverse events (irAEs) incidence is not straightforward. Some studies suggest no appreciable increase in overall toxicity with age; immune checkpoint inhibitors (ICIs) appear relatively safe across age groups [5,6]. However, other research reports higher rates or severity of irAEs in older populations, particularly among those aged 75-84, who experience more treatment disruptions, higher transitions to best supportive care (BSC), and potentially life-threatening complications [7,8] Some very elderly cohorts even show a decreased prevalence of irAEs; however, this may reflect underreporting, milder treatment approaches, or altered immune responses, rather than true resilience [6] 

A biomarker-oriented review suggests that while PD-L1 expression, high tumor mutational burden (TMB), and MCPyV status may influence response, they are not reliable predictors of efficacy in MCC [9]. This is particularly relevant for elderly patients, whose immune profiles may be altered. Nonetheless, the durability of responses in these patients appears preserved, especially when disease burden is low and PS is acceptable. 

In the case we report, despite her advanced age and baseline ECOG PS 3, the patient achieved a significant clinical response with minimal toxicity and a durable benefit that persisted after treatment discontinuation. Due to the resolution of facial disfiguration, her quality of life was also significantly improved. This real-world experience supports previous findings from the literature that favor a patient-centered, not age-centered, approach to immunotherapy decisions in MCC. 

Avelumab may be a feasible and effective therapeutic option in select very elderly patients with advanced MCC [10], offering durable responses and acceptable tolerability. Individualized treatment decisions, considering biological rather than chronological age, remain crucial. Further studies focusing on this growing patient population are warranted. 

## Conclusions

This case illustrates that avelumab can be a safe and effective therapeutic option even in very elderly patients with advanced MCC. Despite advanced age and multiple comorbidities, the patient achieved meaningful and durable disease control with an excellent tolerance profile. This experience reinforces that chronological age alone should not exclude patients from immunotherapy when clinical conditions, performance status, and organ function are adequate. Careful patient selection, multidisciplinary evaluation, and vigilant monitoring for immune-related adverse events are crucial to optimize outcomes in this frail population. This report contributes to the limited real-world literature on immunotherapy use in very elderly individuals with MCC and supports further investigation into predictors of benefit and tolerance to checkpoint inhibitors in this underrepresented age group.
